# Optimising ambulance service contribution to clinical trials: a phenomenological exploration using focus groups

**DOI:** 10.29045/14784726.2019.12.4.3.55

**Published:** 2019-12-01

**Authors:** Helen Pocock, Michelle Thomson, Sarah Taylor, Charles D. Deakin, Ed England

**Affiliations:** South Central Ambulance Service NHS Foundation Trust: ORCID iD: http://orcid.org/0000-0001-7648-5313; South Central Ambulance Service NHS Foundation Trust; South Central Ambulance Service NHS Foundation Trust; South Central Ambulance Service NHS Foundation Trust: ORCID ID: https://orcid.org/0000-0002-2565-9771; South Central Ambulance Service NHS Foundation Trust: ORCID iD: https://orcid.org/0000-0002-8009-2843

## Abstract

**Introduction:**

Out-of-hospital cardiac arrest trials can prove challenging and there is a need to share learning from those that have recruited successfully. We have just completed three years of recruitment to PARAMEDIC2, a placebo-controlled trial of adrenaline in out-of-hospital cardiac arrest. This study was designed to describe the experience of operational ambulance staff involved in recruiting patients into PARAMEDIC2.

**Methods:**

Four focus groups involving trial paramedics and supporting members of the emergency care team were conducted across different geographical regions of a single UK ambulance service participating in the PARAMEDIC2 study. Data analysis was supported by NVivo 12 and themes were identified using a thematic analysis approach.

**Results:**

Forty-four participants contributed to the focus groups. Four overarching themes were identified: *context for the research*, *ethical concerns*, *concerns at the patient’s side* and *ongoing trial support*. Participants felt that research such as PARAMEDIC2 is important and necessary to drive medical progress. They valued the opportunity to be part of a large project. Due to the deferred consent model employed, public awareness of the trial was felt to be important. Most expressed equipoise regarding adrenaline, but some felt concerned about enrolling younger patients and there was discussion around what constitutes a successful outcome. Struggles with ethical concerns were overcome through training and one-to-one discussion with research paramedics. Participants valued feedback on their performance of trial tasks, but also wanted feedback on their resuscitation skills. Cardiac arrest places a high cognitive demand on paramedics; simplicity and reinforcement of trial processes were key to facilitating recruitment. Caring for relatives was a high priority for paramedics and some felt conflicted about not discussing the trial with them.

**Conclusion:**

This study has provided insights into paramedic experience of a large-scale pre-hospital trial. Investment in time and resource to provide face-to-face training and personalised feedback to paramedics can foster engagement and optimise performance.

